# Role of Wnt11 during Osteogenic Differentiation of Human Mesenchymal Stem Cells on Microstructured Titanium Surfaces

**DOI:** 10.1038/s41598-018-26901-8

**Published:** 2018-06-05

**Authors:** Barbara D. Boyan, Rene Olivares-Navarrete, Michael B. Berger, Sharon L. Hyzy, Zvi Schwartz

**Affiliations:** 1 Department of Biomedical Engineering, School of Engineering, Virginia Commonwealth University, Richmond, VA 23284 USA; 2 Wallace H. Coulter Department of Biomedical Engineering, Georgia Institute of Technology, Atlanta, GA 30332 USA; 3 Department of Periodontics, University of Texas Health Science Center at San Antonio, San Antonio, Texas 78229 USA

## Abstract

Successful osseointegration of an endosseous implant involves migration and differentiation of mesenchymal stem cells (MSCs) on the implant surface. Micro-structured, hydrophilic titanium surfaces direct MSCs to undergo osteoblastic differentiation *in vitro*, in the absence of media additives commonly used in cultures grown on tissue culture polystyrene (TCPS). This process involves non-canonical Wnt5a, in contrast to canonical Wnt3a typically credited with osteoblastic differentiation on TCPS. Wnt proteins have been implicated in morphological development and tissue patterning, suggesting that additional Wnts may participate. Here, we demonstrate that Wnt11 is a mediator of osteoblast commitment of MSCs, and increases in a surface-roughness dependent manner. Experiments using cells silenced for Wnt11 indicate that cross-talk between Wnt5a and Wnt11 occurs. Wnt11 potentially acts upstream to Wnt5a, increasing Wnt5a expression and factors associated with osteogenesis. Thus, Wnt11 contributes to peri-implant bone formation distal to the implant surface through a heavily regulated signaling cascade of autocrine/paracrine proteins.

## Introduction

Wingless-related integration site (Wnt) proteins are highly conserved, secreted glycoproteins involved in the regulation of cell proliferation, differentiation, adhesion and migration in many organs and tissues of mammals^1^. The Wnt protein family consists of two major signaling subsets, canonical and non-canonical, defined by their interaction with β-catenin. Canonical Wnt proteins (Wnt1/3/3a/8) activate the Wnt/β-catenin pathway, while non-canonical Wnt proteins (Wnt4/5a/5b/11) activate the Wnt calcium-dependent pathway^2^. Both canonical and non-canonical Wnts behave in an autocrine/paracrine manner. In brief, canonical Wnt signaling increases β-catenin accumulation and translocation into the nucleus. The binding of canonical Wnt proteins to members of the Frizzled receptors (Fzd) and its co-receptors results in the activation of Dishevelled and inhibition of the β-catenin degradation complex, allowing targeting of T-cell specific transcription factor/lymphoid enhancer-binding factor 1 (TCF/LEF) family of transcription factors and transcriptional activation of Wnt target genes^3,4^.

Wnt proteins are key regulators of osteoblast differentiation and maturation^5^. Recent studies have shown that Wnt/β-catenin signaling is crucial for cell proliferation and suppression of osteogenic differentiation in adult human mesenchymal stem cells (MSCs). MSCs cultured on tissue culture polystyrene (TCPS) and treated with Wnt3a exhibit reduced osteogenic differentiation, including decreased matrix mineralization and reduced alkaline phosphatase mRNA and activity^6^. Moreover, osteogenic markers are suppressed in osteoblasts when treated with Wnt3a on TCPS^6^. Conversely, our lab shown that canonical Wnt3a regulated proliferation but had no effect on osteoblast differentiation when MSCs were cultured on titanium (Ti) substrates possessing micro-/nano-roughened surfaces. Furthermore, when Wnt3a signaling was inhibited in MSCs, the amount of bone morphogenetic proteins (BMP2/4) increased^7^, which may have contributed to the rapid increase in osteoblast differentiation seen in MSCs cultured on microstructured Ti substrates, suggesting substrate cues may alter cell response to biochemical cues.

Wnt proteins in the non-canonical calcium-dependent pathway stimulate the release of intracellular Ca^++^ to activate calmodulin-dependent kinase II (CaMKII), protein kinase C (PKC), and calcineurin^8^, resulting in osteoblast differentiation via PKC-dependent signaling in mesenchymal progenitor cells^2,9^. In osteoblasts, stimulation of PKC by Wnt5a requires the participation of protein disulfide isomerase A3 (PDIA3), phospholipase A2 activating protein (PLAA), and CaM^2,10^. We showed previously that Wnt5a promotes osteoblastic differentiation and maturation of MG63 osteoblast-like cells cultured on micro-/nano-roughened surfaces by upregulation of BMP2 and BMP4 proteins^7^. The shift in MG63 cells from proliferation to differentiation on these microtextured substrates occurs within 7 days in the absence of media additives commonly used to induce osteoblast differentiation in cultures grown on TCPS^11,12^. Moreover, osteoblast differentiation is accompanied by morphological maturation caused by non-canonical Wnt signaling^13,14^. Similarly, MSCs exhibit an osteoblast phenotype within 7 days when cultured in growth media on microtextured Ti substrates, whereas this process normally requires 21 days for cells on TCPS, even when osteogenic medium is used^15,16^.

Wnt11 is a secreted glycoprotein with a molecular mass of 45 kDa and associates with the extracellular matrix in many tissues^17–21^. *WNT11* expression increases during glucocorticoid-induced osteogenesis, suggesting a role in osteoblast differentiation on TCPS^6^. Typical of other non-canonical Wnts, Wnt11 acts through the calcium-dependent pathway^22–24^. Conversely, there is evidence that Wnt11 also induces osteoblast differentiation through canonical signaling and enhancing BMP-induced osteoblast maturation and mineralization in pre-osteoblastic cells^25^.

Non-canonical signaling by Wnt11 promotes Daam1 and small GTPases Cdc42 and RhoA, which have been shown to promote tissue regulation signals during vertebrate gastrulation^26,27^. Additionally, *WNT11* mRNA has been shown to be upregulated during adult human embryonic MSC chondrogenesis^28^. Moreover, *WNT11* overexpression has been proven to promote chondrogenesis of bone marrow-derived MSCs by upregulation of chondrogenic regulatory markers, *RUNX2* and Indian Hedgehog^29^. Wnt11 has shown the ability to inhibit canonical Wnt signaling through various intracellular pathways^30^, which further suggests endogenous Wnt regulation by non-canonical signaling, specifically during osteoblast differentiation and chondrogenesis.

Non-canonical Wnt5a and Wnt11 play important roles in osteoblast differentiation, but the function of Wnt11 and the relationship between Wnt11 and Wnt5a remain unclear. In the present study, we examined the role of Wnt11 during MSC differentiation on Ti surfaces with different roughness and energy. We further examined the relationship between Wnt5a and Wnt11, hypothesizing that cross-talk between the two proteins is required for the rapid osteoblast differentiation observed on microtextured Ti substrates.

## Methods

### Material Manufacturing

Grade 2 Ti discs, 15 mm in diameter and 1 mm thick, were manufactured by Institut Straumann AG (Basel, Switzerland) and modified to produce three surfaces: pretreatment (PT), SLA, and modSLA. Manufacturing methodology of these surfaces has been previously described^31^. In brief, PT discs were degreased by washing in acetone, and processed via a 2%ammonium fluoride/2% hydrofluoric acid/10%HNO_3_ acid solution at 55 °C for 30 seconds. PT discs were then modified to produce SLA and modSLA discs. SLA discs were sand-blasted with large grit corundum (250–500 μm), and subsequently acid etched in a hot acid (>100 °C) mixture of HCl and H_2_SO_4_. Discs were then cleaned in HNO_3_ and rinsed in ultrapure water, packed into aluminum foil, and γ-irradiated before use. modSLA discs underwent the same initial modification process as SLA; however, HNO_3_ cleaning and all steps following were conducted in a nitrogen environment to prevent hydrocarbon deposition. The discs were then rinsed and stored in an isotonic NaCl solution and γ-irradiated.

### Material Characterization

The PT, SLA and modSLA surfaces have previously been described in detail^12,31–33^. Prior to use in this study, batch validation was performed. Qualitative analysis of each surface was conducted by scanning electron microscopy (SEM, Hitachi SU-70); samples were imaged at 56 μA ion current, 5 kV accelerating voltage and 4 mm working distance. Representative images from an n = 10 per disc for 3 discs are shown for each group. Surface energy was determined by sessile drop test using a goniometer (CAM 250, Ramé-Hart). Samples (n = 3) were measured in 3 different locations and dried with nitrogen between measurements. 3 μL of ultrapure water was used per drop measurement, and angle measurements were taken every 5 seconds for a total of 20 seconds. Those four measurements were then averaged to produce 1 of the 3 total measurements per disc. Measurements are shown as mean ± SEM of 9 samples per group.

### Cell Culture

Bone marrow derived human MSCs (Lonza, Walkersville, MD) were cultured on TCPS, PT, SLA, or modSLA surfaces at a seeding density of 10,000 cells/cm^2^. MSC growth medium (MSCGM, Lonza) was used to plate and feed cells 24 hours after plating, and every 48 hours thereafter. At confluence, medium was replaced with MSCGM and cultures were allowed to incubate for 12 hours for RNA extraction and analysis, and 24 hours for protein analysis experimentations. For experiments involving treatment of cells with exogenous Wnt5a or Wnt11 as described below, Wnts were added to the cultures at confluence.

### Wnt11 Silencing

Wild-type (WT) bone marrow derived MSCs were transduced with one of five shRNA lentiviral transduction particles (SHCLNV NM_004626, Mission®, Sigma Aldrich, St. Louis, MO) to silence the *WNT11* gene. MSCs were plated at 40,000 cells/cm^2^ in a 6 well plate for 24 hours. Lentiviral particles were added to the cells at a multiplicity of infection of 7.5. After an 18 h incubation, transduced cells were selected with 0.25 μg/ml of puromycin. Silencing of *WNT11* was confirmed at >70% knockdown using the real-time qPCR technique described below (data not shown). Two vectors demonstrated silencing >70% and the vector with greatest effect was chosen. Vectors that showed no silencing were used as controls to assess the effects of silencing on phenotypic expression on TCPS. Cells transfected with these vectors exhibited no phenotypic differences on TCPS compared to wild type (WT) cells. Therefore, WT cells were used as controls for analyses on Ti surfaces.

### Cell Imaging

WT and si*WNT11* MSCs were plated on TCPS at a density of 5,000 cells/cm^2^. At 2, 6, 12, 24 and 48 hours, cell layers were fixed in 4% paraformaldehyde for 20 minutes and permeablized in 0.05% Triton X-100 in PBS for 5 minutes. To visualize F-actin, cells were incubated for 1 hour with Alexa Fluor 488-labeled phalloidin (Life Technologies, Carlsbad, CA). At the end of the incubation period, cells were washed with PBS and incubated with Hoechst 33342 (Invitrogen) for 10 minutes to stain nuclei. Finally, cultures were washed with 0.05% Triton X-100 in PBS, mounted on glass coverslips with Fluoro-Gel with Tris buffer (Electron Microscopy Sciences, Hatfield, PA) and imaged (Zeiss LSM 510 Non-Linear Optics with META Multiphoton Excitation, Carl Zeiss Microscopy, Thornwood, NY).

### Protein Expression

In order to determine if MSCs were responsive to Wnt11, we examined the effects of Wnt11 using WT MSCs cultured on TCPS. At confluence, cells were treated with 50 ng/mL recombinant human Wnt11 (R&D Systems, Minneapolis, MN). At confluence, fresh MSCGM was added to the cultures and conditioned media were collected 24 hours later.

After establishing responsiveness to Wnt11, WT and si*WNT11* MSCs were cultured on TCPS and the Ti disks. 24 hours after plating, the media were changed. Thereafter the cells were treated with either 50 ng/mL recombinant human Wnt11 (R&D Systems) or 50 ng/mL recombinant human Wnt5a (R&D Systems). Cultures were exposed to fresh Wnt proteins at each 48 hours media change. At confluence on TCPS (roughly 7 days), cells on all surfaces were incubated in fresh media without exogenous Wnt proteins for 24 hrs. The conditioned media were collected and osteocalcin (OCN) levels measured by radioimmunoassay (Biomedical Technologies Inc., Stoughton, MA). Levels of osteoprotegerin (OPG) (R&D Systems), VEGF (R&D Systems), TGF-β1 (R&D Systems), BMP2 (*Pepro Tech*, Rocky Hill, NJ) and BMP4 (R&D Systems) in the conditioned media were measured by ELISA per manufacturer’s instructions, as described previously^34,35^. Cells were harvested from the surfaces by two serial trypsinizations and counted. Cells were lysed and alkaline phosphatase specific activity and protein levels were measured in the lysate.

### Gene Expression

To determine whether Wnt pathway genes were regulated during differentiation of MSCs on microstructured Ti substrates, three analyses were conducted. In the first, MSCs were plated on TCPS and cultured until cells reached confluence on TCPS. Cells were incubated with fresh media supplemented with 50 ng/mL Wnt3a, 50 ng/mL Wnt5a, or 50 ng/mL Wnt11 for 12 hrs and harvested using TRIzol (Invitrogen, Carlsbad, CA).

In a subsequent analysis, a time course of expression was determined. MSCs were plated on TCPS or Ti disks, treated with Wnt proteins as above, and were harvested after 2, 4, or 6 days of culture. Cells were incubated with fresh media for 12 h on day of harvest and RNA extracted using the RNAqueous-Micro RNA extraction kit (Applied Biosystems, Carlsbad, CA).

Finally, we determined the effect of Wnt11 on mRNA for integrins known to be differentially expressed in MSCs as a function of surface topography and free energy and shown to be responsible for mediating the effects of surface properties in osteoblast differentiation^36–39^. WT and si*Wnt11* silenced MSCSs were plated on TCPS and Ti discs. At confluence, cells were treated with fresh media for 12 h. RNA was isolated using TRIzol.

For all studies, RNA was quantified using a NanoDrop Spectrophotometer (Thermo Scientific, Waltham, MA). To create a cDNA template, 125 ng of RNA was reverse transcribed using a High Capacity Reverse Transcription cDNA kit (Applied Biosystems). To quantify expression of WNT ligands, receptors, co-receptors, and inhibitors, cDNA was used for real-time PCR with gene-specific primers using the Step One Plus Real-time PCR System and Power Sybr® Green Master Mix (Applied Biosystems). Fluorescence values were quantified as starting quantities using known dilutions of MSCs grown on TCPS. Genes are presented as normalized to GAPDH. Primers (Supplemental Table 1) were designed using the Beacon designer software and synthesized by Eurofins MWG Operon (Huntsville, AL).

### Statistical Analysis

Data are means ± SEM of six independent cultures/variable. Data were analyzed by one way ANOVA, and secondary post-tests for differences between groups were determined using Bonferroni’s modification of Student’s t-test. Significant differences between groups were determined at p < 0.05. All experiments were performed a minimum of two times to ensure validity of the data.

### Disclosures

BDB is a consultant for Institut Straumann AG (Basel, Switzerland) and Titan Spine LLC (Mequon, WI). This study was funded in part by Institut Straumann AG. ZS is a consultant for AB-Dental (Ashdod, Israel).

## Results

### Material Characterization

**Figure 1:**
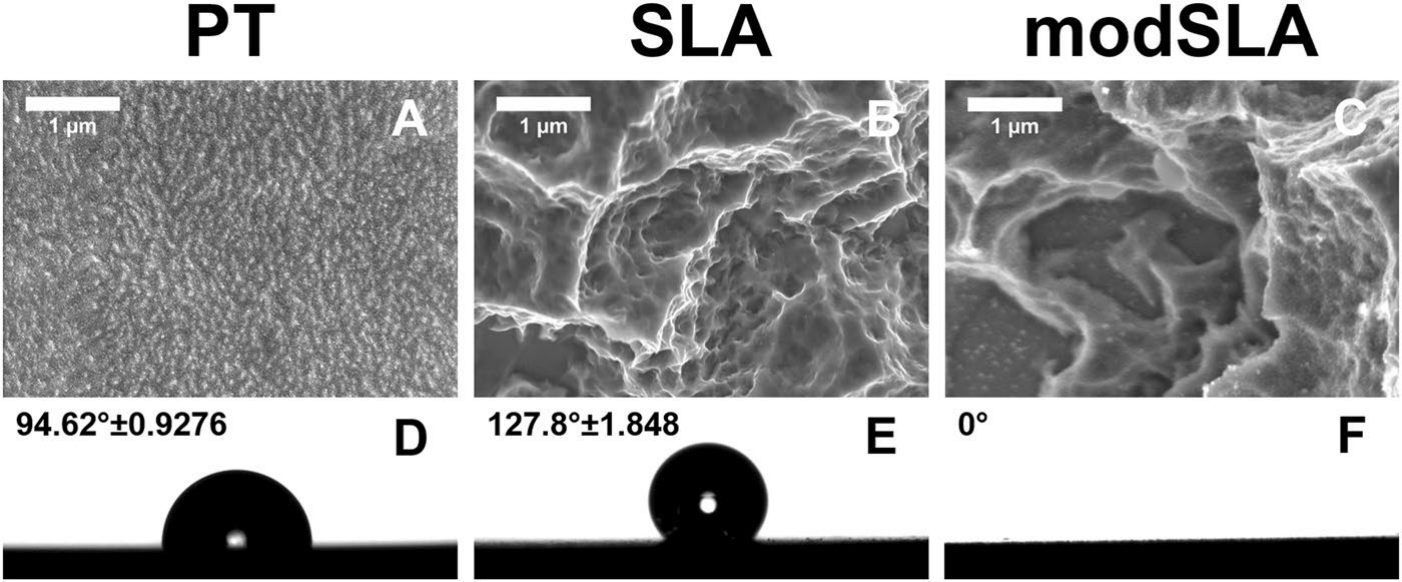
Titanium surfaces are capable of undergoing modifications for micro-/nano-rough surface topography and increased surface energy. 15 mm × 1 mm grade 2 Ti pretreated (PT), grit-blasted and acid-etched (SLA), and hydrophilic SLA (modSLA) surfaces were imaged by scanning electron microscopy (**A**–**C**) at 25kX scale bar is 1 μm. Surface energy was analyzed by sessile drop contact angle measurement (**D**–**F**).

Assessment of surface topography by SEM and laser scanning confocal microscopy demonstrates altered surface morphologies dependent upon the manufacturing protocol. PT surfaces had a flattened morphology with a lack of micro-scale 10–50 μm and 1–2 μm structures (Fig. 1A); while both SLA and modSLA had both surface features (Fig. 1B,C). SEM micrographs qualitatively showed no differences between SLA and modSLA at the micro-scale (Fig. 1B,C), however, small nanostructures could be seen on modSLA (Fig. 1C).

Surface energy determined by sessile drop contact angle measurement demonstrated differences among the substrates. PT surfaces were hydrophobic with 94.6° ± 0.9; SLA surfaces had contact angles of 127.8° ± 1.9 (Fig. 1D,E). modSLA surfaces were super hydrophilic with a contact angle of approximately 0° (Fig. 1F).

### Effect of WNT11 on Osteoblast Gene Expression of MSCs

**Figure 2:**
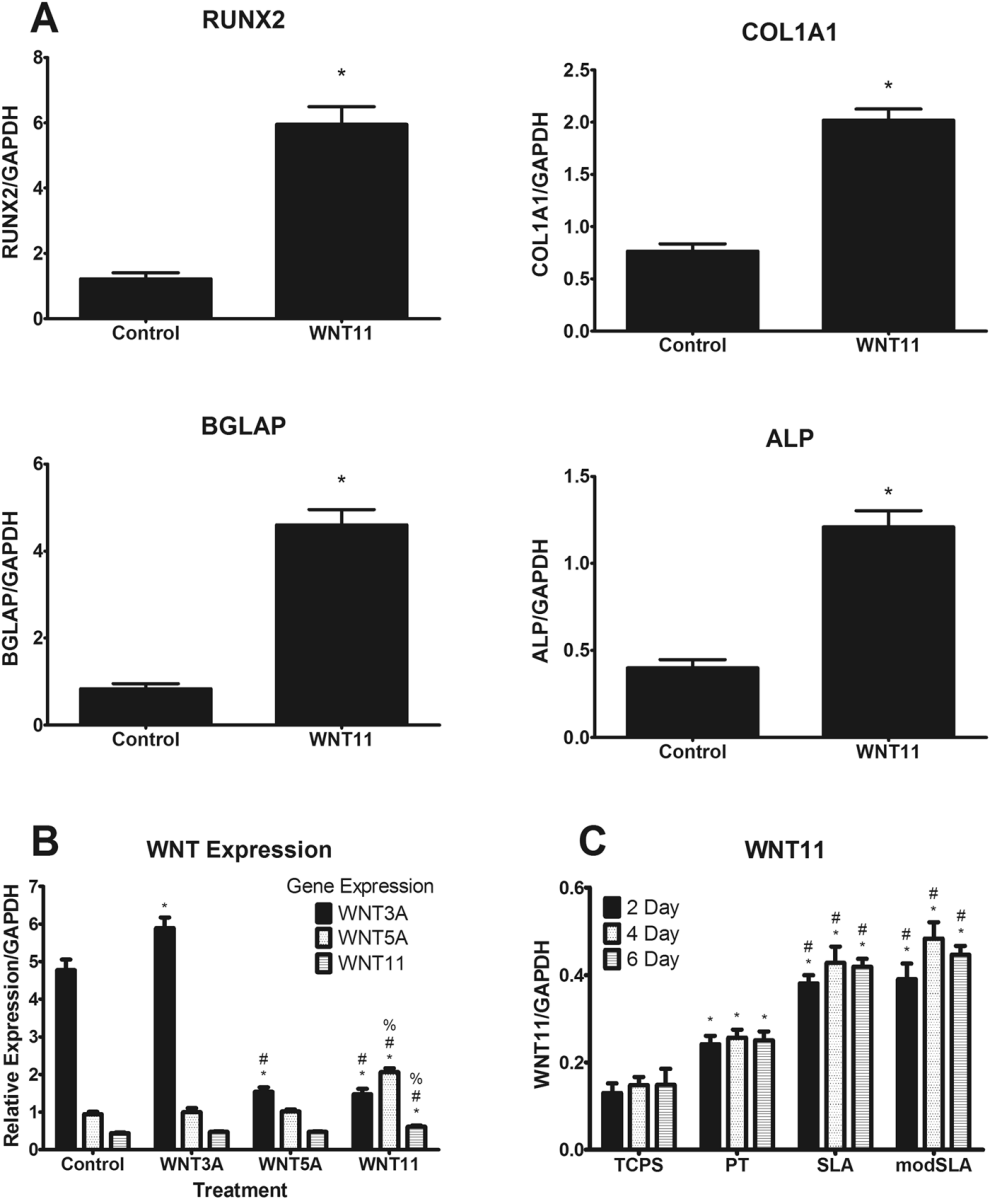
Effects of Wnt treatment on MSCs gene expression. MSCs were cultured to confluence on TCPS and treated with fresh media supplemented with 50 ng/mL recombinant human Wnt11 for 12 hours. RNA was extracted and gene expression analysis was conducted by RT-qPCR for runt-related transcription factor 2 (*RUNX2*), collagen type 1 (*COL1A1*), bone gla-protein (*BGLAP*), and alkaline phosphatase (*ALPL* **A**). Interaction of Wnt treatments on *WNT* gene expression was examined by RT-qPCR for *WNT3A*, *WNT5A*, and *WNT11* (**B**) after treatment for 12 hours with 50 ng/mL of Wnt3a, Wnt5a, or Wnt11. ^*^p < 0.05 vs control; ^#^p < 0.05 Wnt3a treatment; ^%^p < 0.05 vs Wnt5a treatment. RT-qPCR was conducted for *WNT11* for MSCs cultured on TCPS or Ti surfaces for either 2, 4, or 6 days (**C**). ^*^p < 0.05 vs TCPS; ^#^p < 0.05 vs PT.

MSCs cultured on TCPS and treated with Wnt11 exhibited increased expression of osteoblast genes for the transcription factor RUNX2 (*RUNX2)*, type 1 collagen (*COL1A1)*, osteocalcin (*BGLAP)*, and tissue non-specific alkaline phosphatase (TNAP) compared to non-treated control (Fig. 2A).

When MSCs were cultured on TCPS, *WNT* gene expression was differentially regulated by Wnt family proteins (Fig. 2B). Treatment with Wnt3a increased expression of its own gene, but did not alter *WNT5A* or *WNT11* expression compared to control. However, treatment with Wnt5a decreased *WNT3A* expression compared to control and Wnt3a treatment, but generated no changes in *WNT5A* or *WNT11*. Wnt11 was the only treatment to increase expression of both *WNT5A* and *WNT11*, while *WNT3A* was decreased compared to control and Wnt3a treatment.

No time-dependent changes in *WNT11* expression were observed in cultures grown on TCPS or the Ti substrates. However, *WNT11* expression was affected by the substrate. Expression on PT was increased compared to TCPS, and expression on both SLA and modSLA was further augmented compared to PT. There was no difference between SLA and modSLA, indicating that surface energy was not a factor (Fig. 2C).

### Effects of Surface Properties on Regulation of Osteoblast Differentiation by WNT11

**Figure 3:**
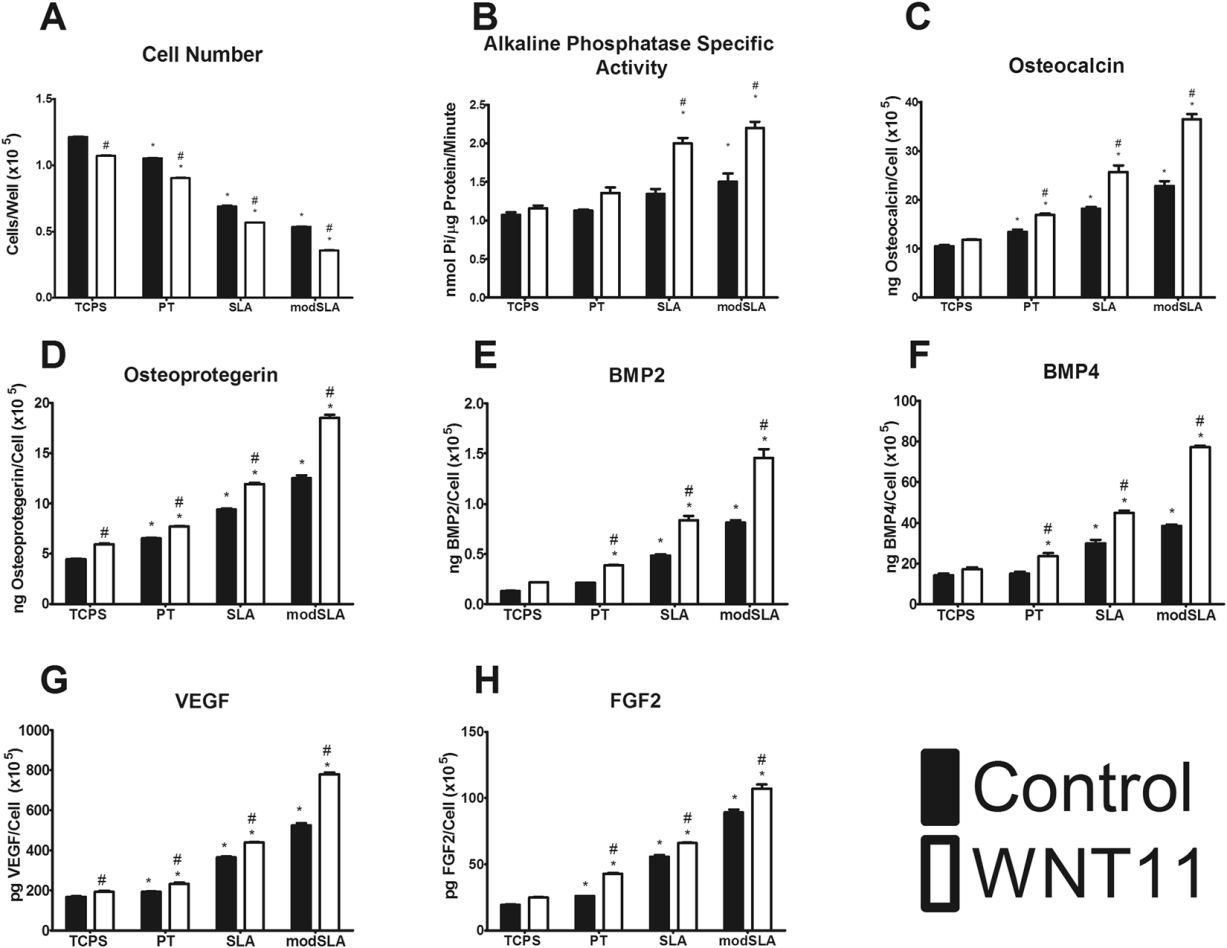
Autocrine & paracrine effects of *WNT11* on Ti surfaces. MSCs were cultured on TCPS, PT, SLA, modSLA till confluence on TCPS and treated with fresh media with or without 50 ng/mL recombinant human Wnt11. At confluence, cell number (**A**), alkaline phosphatase specific activity (**B**), and levels of secreted osteocalcin (**C**), osteoprotegerin (**D**), BMP2 (**E**), BMP4 (**F**), VEGF (**G**), and FGF2 (**H**) were determined. ^*^p < 0.05 vs TCPS; ^#^p < 0.05 vs Control MSCs on each surface.

The effects of Wnt11 on osteoblast differentiation varied with surface properties. Cell number decreased on all Ti substrates compared to growth on TCPS; treatment with Wnt11 caused a further decrease in cell number compared to untreated controls on each surface (Fig. 3A). In the absence of Wnt11, alkaline phosphatase specific activity was only increased on modSLA; Wnt11 had no effect on activity on the smooth PT surface but it stimulated activity on the microstructured SLA and modSLA surfaces (Fig. 3B). Production of both osteocalcin and osteoprotegerin were increased on all Ti substrates compared to TCPS; Wnt11 treatment caused a further increase on the microtextured substrates, with modSLA >SLA (Fig. 3C,D).

Wnt11 had no effect on BMP2 or BMP4 in cultures grown on TCPS but it increased both growth factors on the Ti substrates, with the greatest stimulatory effect on modSLA (Fig. 3E,F). Paracrine signaling factors vascular endothelial growth factor (VEGF) and basic fibroblast growth factor (FGF2) were increased on Ti compared to TCPS (Fig. 3G,H). Wnt11 increased VEGF production by cultures grown on all surfaces with the greatest effect seen in cultures grown on modSLA. FGF2 was not increased by Wnt11 on TCPS; in contrast, Wnt11 increased FGF2 on all Ti substrates.

### Silencing WNT11 Alters Cell Morphology on TCPS

**Figure 4:**
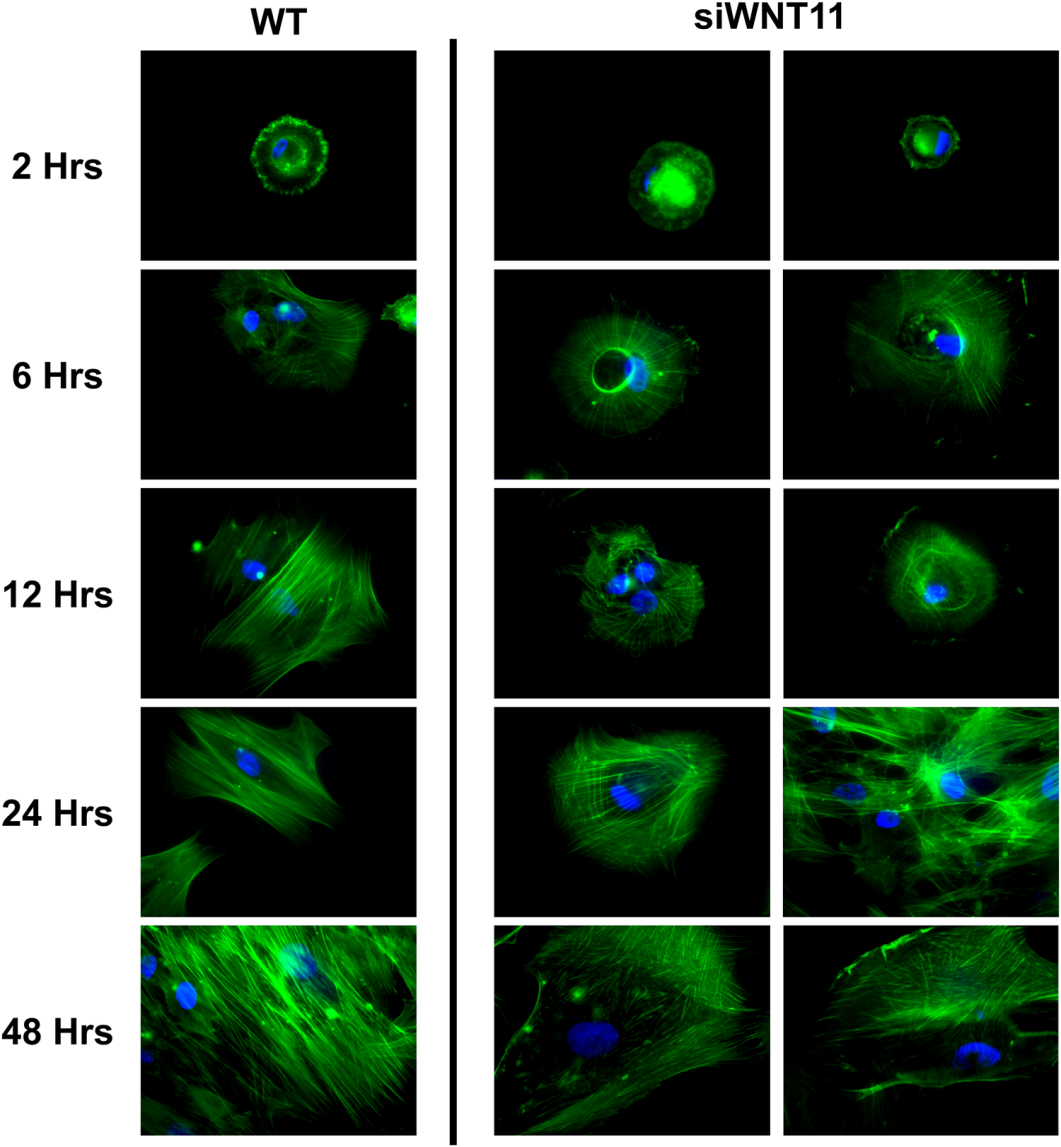
Morphological differences between WT and *WNT11* deficient MSCs. MSCs and si*WNT11* stably transfected MSCs were cultured in chamber slides and fixed at 2, 6, 12, 24 and 48 hours to image cellular attachment and morphological differences. Actin cytoskeleton staining was conducted by Alexa-Fluor 488 (green) labeled phalloidin with the nucleus stained blue by Hoechst 33342. Representative images are shown.

*WNT11* was stably silenced in MSCs (si*WNT11*). Cells with reduced Wnt11 exhibited delayed changes in morphology compared to WT MSCs. At 2 hours, si*WNT11* cells exhibited small diameter circular cellular attachments and cell diameters were smaller than in WT cultures (Fig. 4, 2 Hrs). By 6 hours, development of actin cytoskeleton shifted to more aligned fibers in WT cells while si*WNT11* MSCs remained circular in shape (Fig. 4, 6 Hrs). Further differences were seen at 12 hours with robust actin fiber alignment in WT MSCs; si*WNT11* cells exhibited morphology similar to 6 hour WT MSCs (Fig. 4, 12 Hrs). At 24 and 48 hours, WT cells were fully spread and had centrally located nuclei and increased density (Fig. 4, 24 Hrs, 48 Hrs). si*WNT11* MSCs had impaired actin cytoskeleton development, maintaining a more circular shape through 24 hours and lower density of actin fibers at 48 hours compared to WT cells (Fig. 4, 24 Hrs, 48 Hrs).

### Integrin Expression and Osteoblast Differentiation in si*WNT11* Cells

**Figure 5:**
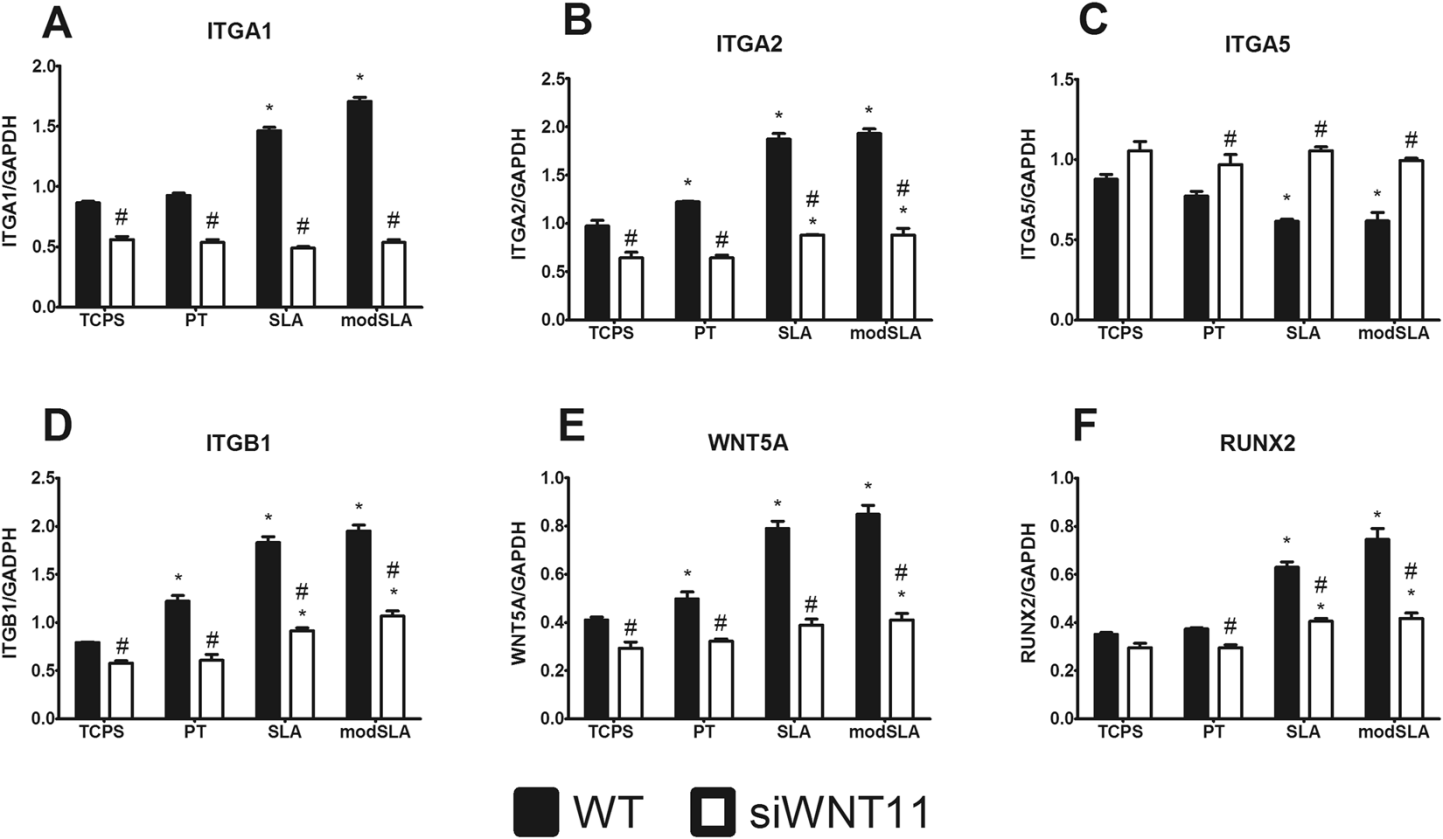
The role of Wnt11 in cell attachment and differentiation of MSCs on Ti substrates. WT and si*WNT11* MSCs were cultured on Ti substrates and TCPS and cultured till confluence. Fresh media was incubated for 12 hours and RNA was extracted. Real-time qPCR was conducted for *ITGA1* (**A**), *ITGA2* (**B**), *ITGA5* (**C**), *ITGB1* (**D**), *WNT5A* (**E**), and *RUNX2* (**F**). ^*^p < 0.05 vs TCPS for each cell type; ^#^p < 0.05 vs Control MSCs on each surface.

Integrins α1β1 and α2β1 have been shown to be the primary receptors and mediators of osteoblastic differentiation on microroughened Ti surfaces^39^. Integrin expression was sensitive to surface properties. In WT cells, *ITGA1* was increased on SLA and modSLA compared to TCPS and PT (Fig. 5A). Expression of integrin α2 (*ITGA2)* was increased on all Ti substrates; this increase was highest on microstructured SLA and modSLA (Fig. 5B). In contrast *ITGA5* was reduced on all Ti substrates with the greatest reduction on SLA and modSLA (Fig. 5C). Similar to *ITGA2*, integrin β1 mRNA (*ITGB1)* was increased on all Ti substrates compared to TCPS and the increase was greatest on SLA and modSLA (Fig. 5D). In si*WNT11* MSCs *ITGA1*, *ITGA2* and *ITGB1* were decreased on all Ti surfaces, but levels of *ITGA2* and *ITGB1* in si*WNT11* cells were still elevated compared to cells on TCPS. Conversely, expression of ITGA5 was restored in control levels in si*WNT11* MSCs on all Ti substrates.

*WNT5A* expression was increased on all Ti substrates compared to TCPS and the increase was greatest on SLA and modSLA (Fig. 5E). Expression was reduced in the si*WNT11* cells by 25% on TCPS. Moreover, in the si*WNT11* cells, expression of *WNT5A* was reduced to levels observed in si*WNT11* cells grown on TCPS. RUNX2 was increased in WT cells grown on SLA and modSLA. This stimulatory effect of the surface was lost in si*WNT11* cells (Fig. 5F).

### Mechanism of WNT11 Effect on MSC Differentiation

**Figure 6:**
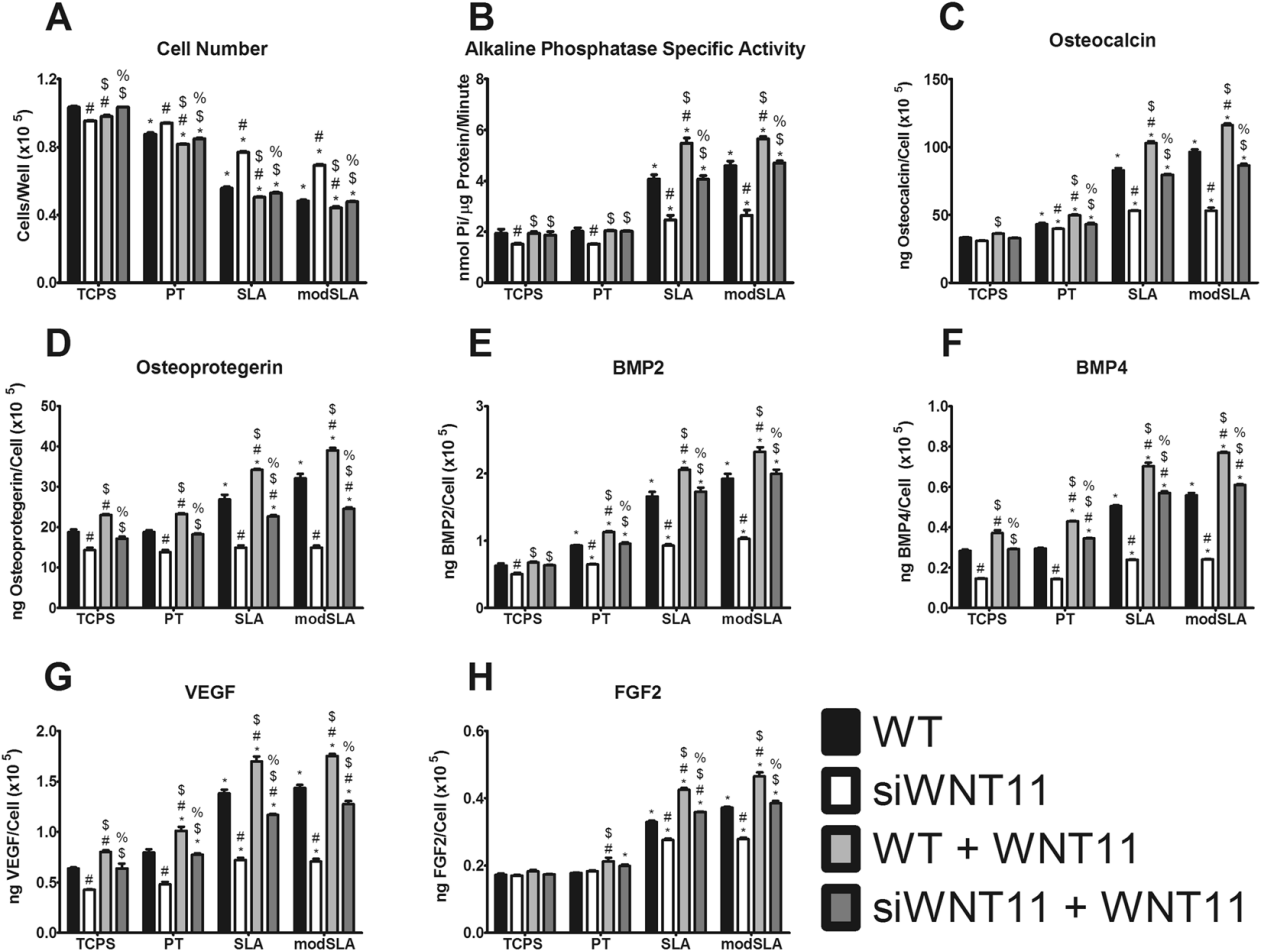
Wnt11 is necessary for differentiation of MSCs on Ti substrates. WT and si*WNT11* MSCs were cultured on TCPS, PT, SLA, and modSLA till confluence receiving media with or without 50 ng/mL of Wnt11. At confluence, cell number (**A**), alkaline phosphatase specific activity (**B**), and levels of secreted osteocalcin (**C**), osteoprotegerin (**D**), BMP2 (**E**), BMP4 (**F**), VEGF (**G**), and FGF2 (**H**) were determined. *p < 0.05 vs TCPS; ^#^p < 0.05 vs WT MSCs on each surface; ^$^p < 0.05 vs si*WNT11* MSCs on each surface; ^%^p < 0.05 vs WT + Wnt11 MSCs on each surface.

Cell number decreased in a surface roughness dependent manner. Silencing *WNT11* prevented the reduction in cell number seen on Ti substrates. Wnt11 treatment decreased cell number compared to cell type controls (Fig. 6A). ALPL activity on TCPS and PT was reduced in si*Wnt11* MSCs compared to WT cells; treatment with Wnt11 only restored the activity of siWNT11 MSCs. Activity was increased on the microstructured surfaces and was further stimulated by Wnt11 compared to WT controls (Fig. 6B).

Osteocalcin and BMP2 were increased on Ti substrates compared to TCPS, and demonstrated a cell type dependence across PT, SLA, and modSLA. These factors were decreased in si*WNT11* MSCs compared to WT cells but were rescued with Wnt11 treatment. Wnt11 supplementation further enhanced WT production due to surface roughness. Treatment with Wnt11 stimulated production of osteocalcin and BMP2 to levels observed in WT cells on the microstructured surfaces (Fig. 6C,E). Osteoprotegerin, VEGF, and BMP4 production were affected in a similar manner but increases were only observed in cultures grown on SLA and modSLA (Fig. 6D,F,G). The effect of surface roughness on FGF2 production was seen only in cells on SLA and modSLA; the surface effect was reduced in siWNT11 cells and this was rescued by Wnt11 treatment (Fig. 6H).

### Interaction of Wnt11 with Wnt5a during Differentiation of MSCs

**Figure 7:**
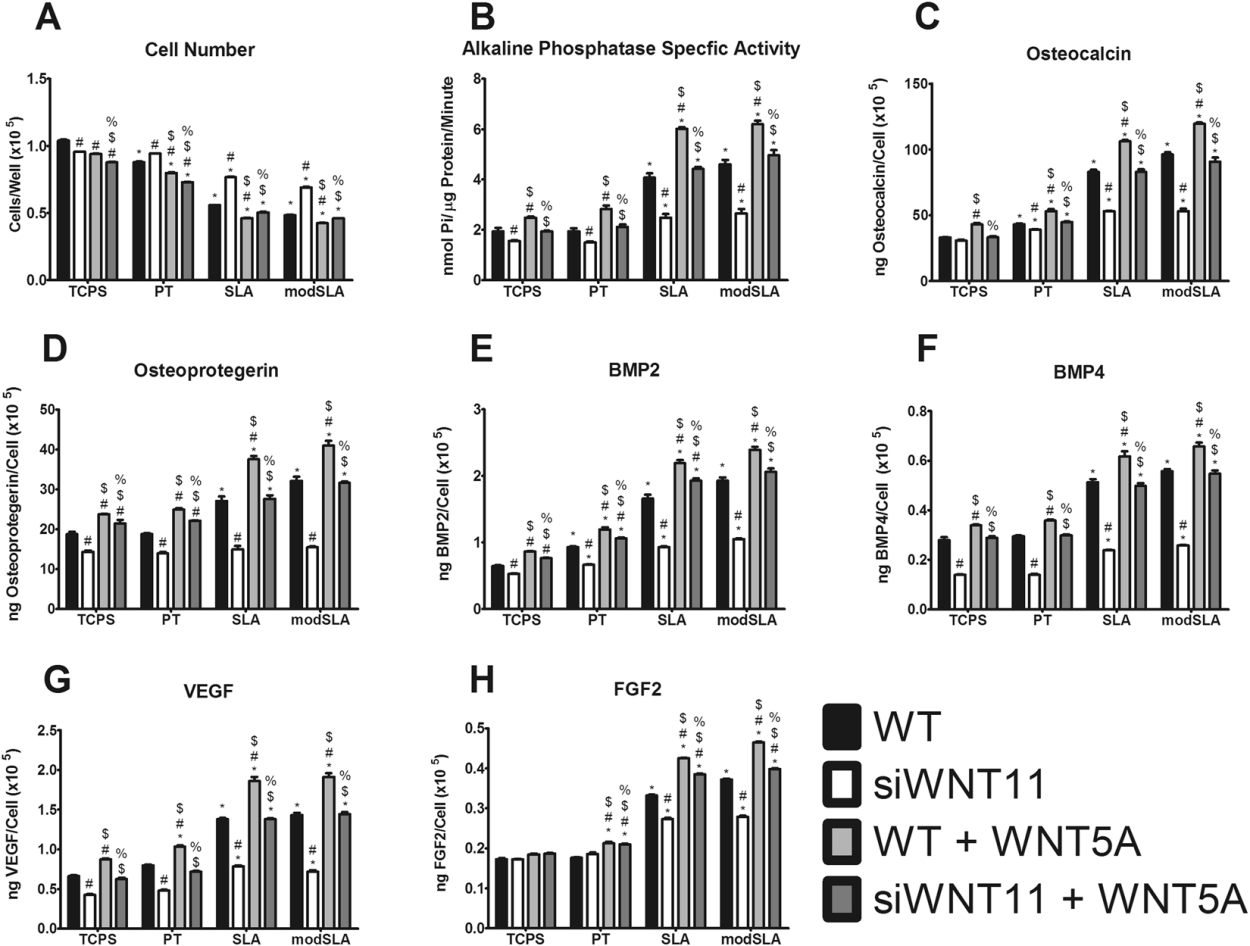
Effects of Wnt5a on WT and si*WNT11* MSCs. WT and si*WNT11* MSCs were cultured on TCPS, PT, SLA, and modSLA till confluence receiving media with or without 50 ng/mL of Wnt5a. At confluence, cell number (**A**), alkaline phosphatase specific activity (**B**), and levels of secreted osteocalcin (**C**), osteoprotegerin (**D**), BMP2 (**E**), BMP4 (**F**), VEGF (**G**), and FGF2 (**H**) were determined. ^*^p < 0.05 vs TCPS; ^#^p < 0.05 vs WT MSCs on each surface; ^$^p < 0.05 vs si*WNT11* MSCs on each surface; ^%^p < 0.05 vs WT + Wnt11 MSCs on each surface.

Wnt5a rescued some, but not all of the effects of *WNT11* silencing. Wnt5a had a stimulatory effect on cell number in WT cells, but it had no effect on cell number in si*WNT11* cells on any surface (Fig. 7A). Wnt5a stimulated ALPL activity in WT and rescued the decrease observed in si*WNT11* cells on all surfaces (Fig. 7B). Wnt5a had a similar effect on osteocalcin, osteoprotegerin, BMP2, BMP4, and VEGF production (Fig. 7C–G). There was no effect of Wnt5a on FGF2 production in WT or si*WNT11* cells grown on TCPS and only a small effect in cells grown on PT. Wnt5a did stimulate FGF2 production by WT cells grown on SLA and modSLA and rescued the reduced FGF2 production observed in si*WNT11* cells on these microstructured surfaces (Fig. 7H).

## Discussion

The present study examined the role of Wnt protein family members in osteoblastic differentiation and maturation on micro-rough Ti surfaces. We previously showed Wnt5a mediated surface effects on differentiation. Here we focused on Wnt11 and the interaction of Wnt5a and Wnt11. The results show a definitive role for Wnt11 in the differentiation of MSCs on modified Ti surfaces possessing micro-roughness and hydrophilicity. Moreover, our results indicate that the surface property involved is topography and not hydrophilicity as there were no differences in cell response to Wnt11 when MSCs were cultured on SLA or modSLA.

It has been suggested that Wnt11 contributes to osteogenic differentiation of MSCs through the stabilization of β-catenin and translocation to the nucleus by canonical Wnt signaling^25^. Our results showing increases in the master transcriptional factor *RUNX2* in MSCs grown on TCPS support this hypothesis, at least for TCPS. Key extracellular matrix components *COL1A1* and *BGLAP* were increased, and *ALPL* expression was increased as well and is corroborated in MC3T3 cells^25^. Our results also show an interesting role for Wnt11 as a potential upstream regulator of Wnt5a production. Wnt11 was the only Wnt to increase expression of Wnt5a. This suggests Wnt11 and Wnt5a may cooperate to activate both canonical and non-canonical signaling pathways. In addition, they may act downstream as a negative feedback mechanism for Wnt3a expression as both proteins decrease Wnt3a production.

Like Wnt5a, Wnt11 contributes to osteoblastic differentiation and autocrine/paracrine signaling of MSCs on Ti substrates. Cell number is reduced on microtextured surfaces and the reduction is correlated with increased production of osteocalcin, indicating osteoblast differentiation^38^. Wnt11 treatment of MSCs decreased cell number in addition to the surface effect, and markedly increased TNAP activity, as well as production of osteocalcin. The effect of Wnt11 on osteoblast differentiation may be a consequence of its stimulatory effect on BMP2 and BMP4 production in MSCs on microtextured Ti surfaces. Moreover, expression of a key regulator of osteoblast differentiation, *RUNX2* was decreased in si*WNT11* cells on all Ti substrates.

Wnt11 treatment also enhanced production of osteoprotegerin, particularly in MSCs grown on micro-rough substrates. Osteoprotegerin is involved in modulating osteoclastic activity, thereby potentially modulating the rate and extent of bone remodeling *in vivo*. Recent studies in our lab confirm that factors present in conditioned media from MSC cultures grown on SLA and modSLA inhibit osteoclast activity *in vitro*^40^. Finally, Wnt11 had a stimulatory effect on VEGF and FGF2 production, suggesting that it may act to promote vascularization of primary bone during osseointegration. Others have shown that proliferation of pre-osteoblasts was stimulated by VEGF and FGF2^41^, further enhancing peri-implant bone formation *in vivo*. Importantly, effects of surface roughness on cell response were lost when *WNT11* was silenced. Wnt11 either had no effect or enhanced the effect of surface topography on WT cells and it restored the effect of surface topography on *WNT11*-silenced cells.

Wnt11 has been shown to be involved in morphological changes in cell shape^42^. Our data confirm the importance of Wnt11 as a regulator of cell shape. Cytoskeletal analysis of si*WNT11* MSCs showed that morphological changes associated with attachment and spreading were delayed compared to WT MSCs. Silenced cells were still capable of developing the morphology of WT MSCs, albeit delayed, suggesting alternative signaling pathways may be involved. Moreover, treatment of *siWNT11* MSCs with Wnt11 was shown to rescue the phenotypic response of the MSCs to WT levels. We hypothesized that Wnt11 works through non-canonical Ca^++^ dependent signaling mediated by integrins. To test this, we examined the expression of integrin subunits previously shown to be involved with differentiation of MSCs on SLA and modSLA^36–39,43^. As expected, α1, α2, and β1 integrins, known for mediating osteoblastic differentiation of MSCs on microtextured Ti, were increased on Ti substrates, and silencing *WNT11* reduced expression on all surfaces. These data suggest cross-talk between non-canonical Wnt11 signaling and expression of key integrin differentiation mediators. Conversely, integrin α5, which functions to negatively regulate osteoblastic differentiation, was increased in si*WNT11* MSCs compared to WT cells.

Our results indicate that Wnt11 is a potential upstream regulator for Wnt5a. However, to definitely determine the temporal regulation of Wnt11 and Wnt5a, studies examining of *siWNT5A* MSCs treated with or without WNT11 need to be conducted. Wnt5a downregulated *WNT3A* but had no effect on *WNT5A* or *WNT11*. Wnt11 also downregulated *WNT3A* but it also upregulated *WNT5A*. Wnt11 increased *WNT5A* on SLA and modSLA and this effect was lost when *WNT11* was silenced. Wnt5a was also able to rescue si*WNT11* cells grown on SLA and modSLA, suggesting redundancy in the role of these two non-canonical Wnts with respect to osteoblast differentiation. While expression of Wnt5a is modulated by Wnt11, it is possible that Wnt5a contributes to the overall change in morphology as an initiator.

Previously, our lab showed the importance of Wnt5a via a Wnt-integrin feedback loop^14,38^. Wnt11 regulates integrin expression in a similar manner. Both Wnt11 and Wnt5a decreased cell number even in si*WNT11* MSCs, supporting the hypothesis that Wnt5a is downstream of Wnt11 and is capable of continuing the differentiation process in the absence of *WNT11* expression in MSCs. Exogenous Wnt11 was capable of increasing TNAP activity and differentiation markers while also contributing to paracrine signaling required for angiogenesis and proliferation. In silenced MSCs, Wnt11 restored production of soluble factors to WT, demonstrating that *WNT11* transcription was not required. The observation that exogenous Wnt5a elicited similar effects suggests that downstream signal recovery occurred either through bypassing Wnt11 or co-activating Fzd receptors that were inactivated during *WNT11* silencing. WT and si*WNT11* MSCs both increased production with Wnt5a treatment to similar levels of Wnt11 treatment.

There are possible implications of Wnt inhibitors on the overall contribution of Wnt11 to osteoblastic differentiation, however it has not been examined. Studies have been conducted regarding Wnt5a with respect to inhibitors Dickkopf-1 (Dkk-1) and Dkk-2^14^. It is probable that Dkk-2 could impact the mechanism of differentiation by Wnt11 due to its effect on later non-canonical calcium-dependent signaling and cross-talk between Wnt5a and Wntt11. Additional evidence in Xenopus models show Wnt5a and Wnt11 act together downstream of Dkk-1 to control dorsal axis formation^44^. Further experimentation is necessary regarding the impact of Wnt modulators and downstream signals on the contribution of Wnt11 during osteoblastic differentiation and is currently being evaluated for future publication. It has been shown that Wnt5a and Wnt11 have downstream signaling effects that can impact overall osteoblastic maturation^7,25^, and as demonstrated in this study by increased osteocalcin, after treatment with both Wnt11 and Wnt5a. Therefore, it is important to examine the impact of Wnt11 and Wnt5a at later time points compared to within the first 7 days as presented in this study, with additional emphasis on downstream targets of both Wnt5a and Wnt11.

## Conclusion

Our findings indicate that differentiation of MSCs mediated by micro-roughened Ti substrates is mediated by Wnt11. Wnt11’s effects are in response to surface topographical clues and not surface hydrophilicity. Our data demonstrate that Wnt11 exhibits cross talk with Wnt5a and is an important regulator of osteoblast maturation involved in primary bone formation during osseointegration. Moreover, Wnt11 mediates the effect of surface topography on new bone formation and its remodeling by affecting the production of factors that modulate osteoclast activity as well as angiogenesis. This effect is most likely controlled by autocrine and paracrine signaling within the Wnt ligand, Fzd receptor, and co-receptors families to produce a heavily regulated signaling cascade to control bone formation necessary for proper osseointegration surrounding an endosseous implant.

## Electronic supplementary material

Supplementary Table 1
