# Supplementary material for: Role of Wnt11 during Osteogenic Differentiation of Human Mesenchymal Stem Cells on Microstructured Titanium Surfaces
Source: Sci Rep. 2018 Jun 5;8:8588. doi: 10.1038/s41598-018-26901-8 (PMC5988747; doi:10.1038/s41598-018-26901-8)
Supplement: Supplementary file 1 — Supplementary Table 1 [file 41598_2018_26901_MOESM1_ESM.pdf]

**Journal:** Scientific Reports

**Title:** Role of Wnt11 during Osteogenic Differentiation of Human Mesenchymal Stem Cells on Microstructured Titanium Surfaces

**Authors:** Barbara D. Boyan[1,2,*], Rene Olivares-Navarrete[1], Michael B. Berger[1], Sharon L. Hyzy[1], Zvi Schwartz[1,3]

**Affiliations:**

[1]Department of Biomedical Engineering, School of Engineering, Virginia Commonwealth University, Richmond VA 23284, USA

[2]Wallace H. Coulter Department of Biomedical Engineering, Georgia Institute of Technology, Atlanta, GA 30332, USA

[3]Department of Periodontics, University of Texas Health Science Center at San Antonio, San Antonio, Texas 78229, USA

**SUPPLEMENTAL INFORMATION**

Supplemental Table 1. Primer sequences used for Real-time PCR analysis of gene expression.

| Gene | Primer Sequence | | Accession Number |
|---|---|---|---|
| GAPDH | F | GCT CTC CAG AAC ATC ATC C | NM_002046.3 |
| | R | TGC TTC ACC ACC TTC TTG | |
| RUNX2 | F | GTC TCA CTG CCT CTC ACT TG | NM_001024630 |
| | R | CAC ACA TCT CCT CCC TTC TG | |
| COL1A1 | F | GAC CTC TCT CCT CTG AAA CC | NM_000088.3 |
| | R | AAC TGC TTT GTG CTT TGG G | |
| ALP | F | TGT GGA GTA TGA GAG TGA CG | NM_000478.3 |
| | R | GAA GTG GGA GTG CTT GTA TC | |
| BGLAP | F | GTG ACG AGT TGG CTG ACC | NM_199173 |
| | R | TGG AGA GGA GCA GAA CTG G | |
| ITGB1 | F | ATT ACT CAG ATC CAA CCA C | NM_002211 |
| | R | TCC TCC TCA TTT CAT TCA TC | |
| WNT 3A | F | GTC CCG TCC CTC CCT TTC | NM_033131 |
| | R | ACC TCT CTT CCT ACC TTT CCC | |
| WNT5A | F | TCT CAG CCC AAG CAA CAA GG | NM_003392 |
| | R | GCC AGC ATC ACA TCA CAA CAC | |
| WNT11 | F | GCT GTA TGA AAT AAT GCT GAG TGA | NM_004626.2 |
| | R | GCC GAG TTC ACT TGA CGA G | |

| | | | |
|---|---|---|---|
| ITGA1 | F | CACTCGTAAATGCCAAGAAAAG | NM_181501.1 |
| | R | TAGAACCCAACACAAAGATGC | |
| ITGA2 | F | ACT GTT CAA GGA GGA GAC | NM_002203 |
| | R | GGT CAA AGG CTT GTT TAG G | |
| ITGA5 | F | ATC TGT GTG CCT GAC CTG | NM_002205 |
| | R | AAG TTC CCT GGG TGT CTG | |
